# Effect of Healthy and Tumor-Associated Breast Adipose Tissue on Breast Cancer Cell Migration and Activation

**DOI:** 10.3390/cancers18050868

**Published:** 2026-03-08

**Authors:** Iris L. Holt-Kedde, Hetty Timmer-Bosscha, Frank A. E. Kruyt, Wendy Kelder, Bert van der Vegt, Mieke C. Zwager, Carolien P. Schröder, Marlous Arjaans

**Affiliations:** 1 Department of Plastic Surgery, University of Groningen, University Medical Center Groningen, 9713 GZ Groningen, The Netherlands; 2 Department of Medical Oncology, University of Groningen, University Medical Center Groningen, 9713 GZ Groningen, The Netherlands; h.timmer-bosscha@umcg.nl (H.T.-B.); f.a.e.kruyt@umcg.nl (F.A.E.K.); 3 Department of Surgery, Martini Hospital Groningen, 9728 NT Groningen, The Netherlands; w.kelder@mzh.nl; 4 Department of Pathology, University of Groningen, University Medical Center Groningen, 9714 GZ Groningen, The Netherlands; b.van.der.vegt@umcg.nl (B.v.d.V.); m.c.zwager@umcg.nl (M.C.Z.); 5 Department of Medical Oncology, Netherlands Cancer Institute, 1066 CX Amsterdam, The Netherlands; c.schroder@nki.nl; 6 Department of Plastic Surgery, OLVG Medical Center, 1061 AE Amsterdam, The Netherlands; m.arjaans@umcg.nl

**Keywords:** adipose tissue, breast cancer, BRCA, obesity, conditioned medium

## Abstract

Background: Obesity is a recognized risk factor for developing breast cancer (BC), but factors involved remain unclear. We investigated if breast adipose tissue from healthy women, BRCA1/2 mutation carriers and BC patients, can stimulate BC cell line migration and activation. Methods: adipose tissue conditioned medium (ATCM), was prepared from breast adipose tissue from healthy subjects (naïve; group 1 (*n* = 20)), BRCA1/2 mutation carriers (group 2 (*n* = 22)) and BC patients (group 3 (*n* = 38)). ATCM effect on migration of BC cell lines MCF-7, SK-BR-3 and MDA-MB-231 was measured with xCELLigence (ACEA Biosciences, San Diego, CA, USA) cell migration assay. Activation of migration was determined by measuring filopodia activation. Migration and filopodia activation were related to body mass index (BMI) and BC subtypes. Luminex multiplex assay was performed to examine the secretory profile of adipose tissue. Results: ATCM from group 1 induced migration and filopodia activation in MCF-7 and MDA-MB-231, but not in SK-BR-3. ATCM from group 2 induced filopodia activation but no migration. ATCM from group 3 induced less migration in MCF-7 than ATCM from group 1. Higher BMI was associated with increased ATCM-induced activation in MCF-7 (group 1) and MDA-MB-231 (group 2). ATCM from group 1 and 2 showed a metabolic secretory profile, whereas group 3 showed higher pro-angiogenic and inflammatory cytokines. Conclusions: This study shows that breast adipose tissue from healthy women, BRCA1/2 mutation carriers and BC patients, can stimulate BC cell line migration and activation. This effect is related to BC subtype and BMI. These data improve insight in adipose tissue as factor in BC development.

## Simple Summary

Breast fat tissue was once considered as only an energy source, but it actively releases signals that can influence nearby cells. Because obesity is linked to breast cancer risk and fat is increasingly used for breast reconstruction, we studied how breast fat tissue affects breast cancer cell behavior. We examined fat tissue from healthy women, women with inherited breast cancer risk genes, and breast cancer patients, and tested how it influenced breast cancer cell movement and activation in the laboratory. Importantly, we found that even healthy breast fat tissue can stimulate cancer cell movement, depending on the cancer type and body weight. This finding is especially relevant because the use of a woman’s own fat for breast reconstruction is increasing. Our results highlight the need for continued safety evaluation of fat-based reconstruction techniques to ensure long-term oncological safety.

## 1. Introduction

Breast cancer (BC) remains the leading cause of cancer-related mortality among women in developing countries and the second leading cause in developed countries [1]. Despite substantial advances in screening and therapy, a significant number of patients still develop locoregional recurrences and distant metastases, resulting in poor long-term outcomes. One of the established risk factors for BC is obesity. Women with obesity have a higher likelihood of developing BC, along with increased risks of locoregional recurrence, distant metastasis, and greater mortality from both BC and other causes [2,3,4,5,6].

During weight gain, adipose tissue undergoes systemic and local changes, including adipocyte hypertrophy, altered metabolism, and low-grade chronic inflammation [7,8,9,10]. Adipose tissue is no longer seen solely as an energy source, but as a metabolically active organ that secretes a wide range of cytokines, chemokines, adipokines, and hormones. Adipocytes in obese individuals may develop a pro-inflammatory and pro-tumorigenic secretory profile capable of influencing nearby epithelial or pre-malignant cells through paracrine signaling, thereby creating chances for tumor cells to flourish [11]. In the presence of BC cells, cancer associated adipocytes can contribute to tumor growth and local invasion [9,12,13,14,15,16,17,18]. Whether also naïve adipocytes (without previous exposure to BC) may exhibit BC subtype specific stimulation, particularly under obese conditions, remains insufficiently understood. In addition, some literature suggests that aromatase expression and cytokine production are different in BRCA1/2 mutation patients and may alter the adipose microenvironment [19,20].

Therefore, we investigated in an in vitro setting if breast adipose tissue from healthy women, BRCA1/2 mutation carriers and BC patients can stimulate BC cell line migration and activation, related to BC subtype and BMI.

## 2. Materials and Methods

### 2.1. Patient Inclusion and Tissue Collection

**Figure 1:**
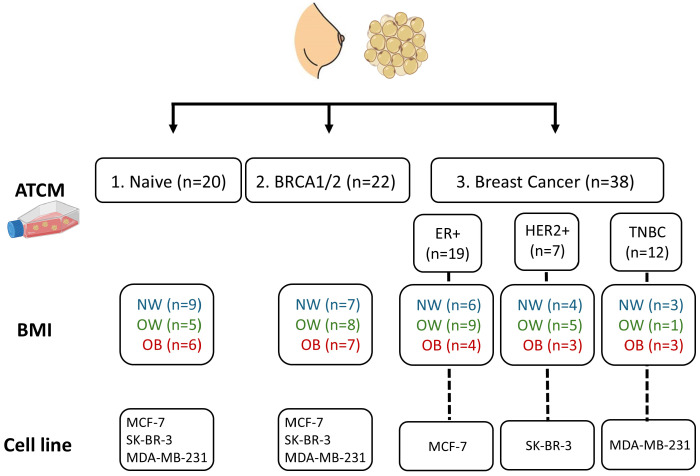
An overview of the different study groups. ATCM (adipose tissue conditioned medium), NW = normal weight (BMI < 25), OW = overweight (25 ≤ BMI < 30). OB = obese (BMI ≥ 30).

Patients were recruited from Academic Breast Center Groningen (location University Medical Center Groningen (UMCG) and Martini Hospital Groningen (MZH)). This study was approved by the Local ethics Review Board and all patients signed informed consent before inclusion (location UMCG number 202000066, approved on 18 May 2020 and location MZH number 2023-025. Approved on 22 February 2023)). Women undergoing elective breast surgery, including reduction mammoplasty, (preventive) mastectomy, or lumpectomy, were asked for permission to collect residual breast adipose tissue as per institutional care protocols. Adipose tissue was transported from the surgical suite in sterile phosphate-buffered saline (PBS) and divided into three study groups: 1. Adipose tissue from subjects undergoing breast reduction surgery (hereafter referred to as naïve adipose tissue), 2. Adipose tissue from BRCA1/2 gene mutation carriers (hereafter referred to as BRCA 1/2 adipose tissue) and 3. Adipose tissue from BC patients. Group 3 was further divided according to BC subtype (estrogen receptor-positive, ER+; human epidermal growth factor receptor 2-overexpressing, HER2+; or triple-negative breast cancer, TNBC, lacking ER, PR, and HER2 expression, as these breast cancer subtypes differ not only in receptor expression but also in their behavior, treatment strategies, and overall prognosis. Each study group was also subdivided according to BMI; normal weight (BMI ≥ 18.5 and < 25), overweight (BMI ≥ 25 and < 30) or obese (BMI ≥ 30). Clinical data regarding age, body mass index (BMI), BC subtype, BRCA1/2 gene mutation status, and history of neoadjuvant therapy were collected from the electronic patient files. Patients with metastatic or recurrent disease or a history of systemic therapy for cancer other than BC were excluded. An overview of the study groups in the different experimental settings is given in Figure 1.

### 2.2. Human Adipose Tissue Conditioned Medium

Preparation of Human ATCM was based on previous description by Wan et al., with some modifications [21]. Fresh adipose tissue samples (up to four per patient) were obtained per patient by the department of Pathology and processed within 1–2 h after collection. After washing and removal of non-adipose components, tissue fragments were cultured in DMEM-Low (Invitrogen, Carlsbad, CA, USA) supplemented with 1% fetal calf serum (FCS) and 1% penicillin-streptomycin at 37 °C and 5% CO_2_ for 48 h under sterile conditions.

After incubation, ATCM was collected, centrifuged to remove residual debris, and aliquoted into sterile 1 mL cryogenic vials. All aliquots were stored at −80 °C until further use in functional assays. A detailed description of the preparation protocol is provided in Supplementary Materials Method S1 and Figure S1.

### 2.3. BC Cell Lines

Three human BC cell lines representing different BC subtypes were used. MCF-7, (ER-positive), SK-BR-3 (HER-2 overexpressing) and MDA-MB-231 (triple negative) were obtained from the American Type Culture Collection (ATCC, Manassas, VA, USA). Cell lines were cultured in DMEM-Low supplemented with 10% FCS and grown at 37 °C in a humidified atmosphere containing 5% CO_2_.

Upon reaching approximately 80% confluency, cells were starved for 24 h in DMEM containing 1% FCS before experimental procedures. All cell lines were routinely tested for Mycoplasma contamination, and their identity was confirmed by short tandem repeat profiling (BaseClear; Leiden, The Netherlands).

### 2.4. Migration Assay Using Real-Time Cell Analysis

Real-time monitoring of BC cell migration was performed using the xCELLigence RTCA DP system (Agilent Technologies, Santa Clara, CA, USA) with a CIM-plate 16 inserts. Cells (5 × 10^4^ cells/well) were seeded in the upper chamber in DMEM-Low with 1% FCS. The lower chamber was filled with ATCM from the three study groups. Negative (1% FCS) and positive (20% FCS) controls were included in each assay. All samples were tested in triplicate.

Migration was monitored every 10 min for up to 60 h and expressed as corrected cell index (CI). Based on time-course analysis, 36 h was identified as the optimal time point for comparison. Detailed technical specifications and CI correction formulas are provided in Supplementary Materials Method S2 and Figure S2.

### 2.5. Filopodia Activation Assessment Using Scratch Assay

Cells were seeded on coverslips in 24-well plates and allowed to reach approximately 70% confluence. For MCF-7 cells, coverslips were coated with poly-L-lysine to maintain a confluent layer. After 24 h starvation, a scratch was created using a 1 mL pipette tip and cells were treated with 400 µL ATCM from groups 1–3 (group 3 matched to the BC subtype). Each experiment included negative (DMEM-Low with 1% FCS) and positive controls (DMEM-Low with 20% FCS).

After 24 h, cells were fixed with 4% methanol-free formaldehyde, permeabilized with 0.1% Triton, and stained for F-actin using fluorescent phalloidin. Nuclei were counterstained with DAPI. Slides were analyzed using an Axio Imager 2 microscope (Zeiss, Oberkochen, Germany).

**Figure 2:**
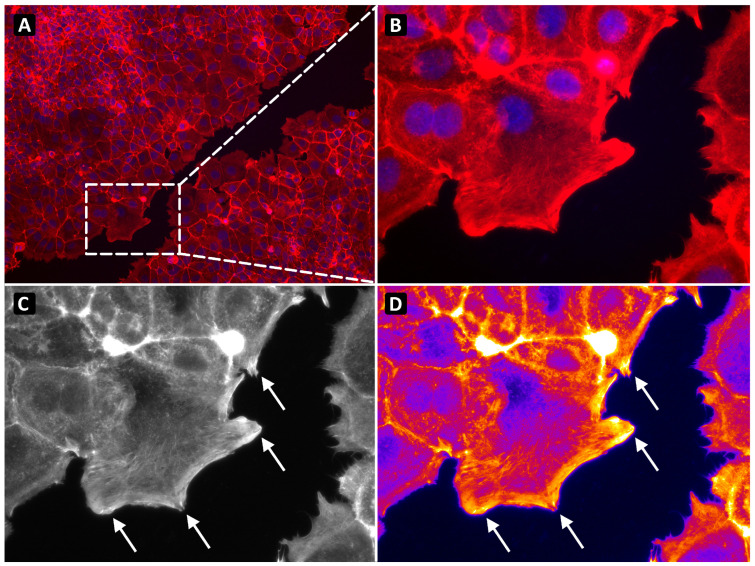
Representative fluorescent images of cells showing filopodia activation in a scratch assay after 24 h. (**A**), overlay image of the scratch with cells stained with DAPI nuclei staining and F-actin staining (magnification 100×); (**B**), example of HPF (high power field) showing filopodia (magnification 400×); (**C**,**D**), Greyscale and Fire lookup table view image to identify activated filopodia (magnification 400×). The arrows indicate activated filopodia.

Filopodia were visualized along the scratch edge and quantified using Fiji ImageJ ( version 2.14.0). Activation was assessed based on phalloidin staining intensity and visualized using the Fire lookup table (Figure 2). Filopodia activation was reported as the percentage of activated filopodia relative to the total number of filopodia.

Detailed staining procedures, imaging parameters, field selection strategy, and manual identification criteria are provided in Supplementary Materials Method S3.

### 2.6. Identifying Cytokines Using Luminex Assay

All 80 ATCM samples were analyzed using Luminex^®^ multiplex immunoassays; Human Obesity (Catalog #FCSTM08 R&D Systems, Minneapolis, MN, USA) and XL Cytokine Premixed Panels (Catalog #FCSTM18B R&D Systems, Minneapolis, MN, USA), R&D Systems. Standards and samples were prepared according to kit instructions, with appropriate dilutions (1:2) made directly in 96-well plates. Microparticle cocktails were added and incubated at room temperature, followed by overnight incubation at 4 °C. The next day, plates were washed and sequentially incubated with biotinylated detection antibodies and Streptavidin-PE. Fluorescence was measured using a Luminex® 200 analyzer (Luminex Corporation, Austin, TX, USA), and analyte concentrations were determined from standard curves.

## 3. Results

### 3.1. Patients’ Characteristics and Human Breast Adipose Tissue Samples

Breast adipose tissue was collected from 80 patients in total, with 20, 22 and 38 samples for group (1) naïve, (2) BRCA1/2 and (3) BC, respectively. Detailed clinicopathological information is summarized in Table 1. Mean BMI was highest in ER+ BC patients (32.4 ± 3.3) and lowest among healthy individuals (26.7 ± 4.3). Patients with HER2+ BC were oldest (age 58 ± 15 years), whereas subjects with BRCA gene mutation were the youngest (33 ± 14 years). Neo-adjuvant treatment was administrated to 30.7% of TNBC and 14.3% of HER2+ patients (Table 1).

#### 3.1.1. Group 1—Naïve Adipose Tissue (Naïve ATCM)

**Figure 3:**
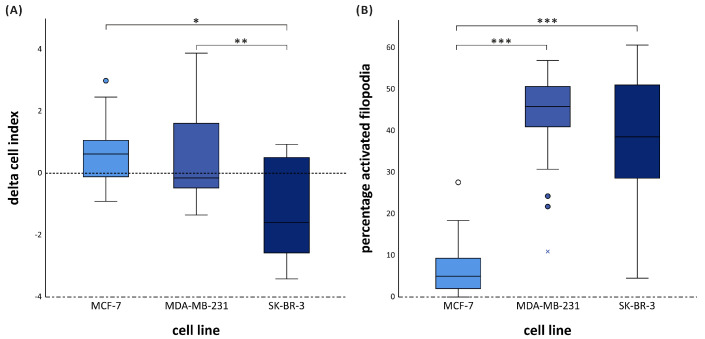
Group 1. (**A**) Migration of MCF-7, MDA-MB-231 and SK-BR-3 cells with naïve adipose tissue derived ATCM as attractant. Delta cell index is corrected for positive and negative control. (**B**) percentage activated filopodia by naïve adipose tissue derived ATCM. Naïve adipose tissue induced migration in MCF-7 and MDA-MB-231 cells (**A**), with stronger filopodia activation in MDA-MB-231 (**B**). (* *p* < 0.005, ** *p* = 0.001, *** *p* < 0.001).

Naïve ATCM affected both migration and filopodia activation in a cell line-dependent manner. For migration, naïve ATCM stimulated motility in MCF-7 and MDA-MB-231 cells, whereas it inhibited migration in SK-BR-3 cells (H (2) = 13.5, *p* = 0.001) (Figure 3A). BMI modified these effects only in SK-BR-3 cells, where obese naïve ATCM further enhanced the inhibitory response compared to overweight naïve ATCM (H (2) = 7.109, *p* = 0.029) (Figure S3A). Naïve ATCM also induced filopodia activation in all three cell lines (H (2) = 34.4, *p* < 0.001). Activation was significantly lower in MCF-7 cells compared with MDA-MB-231 and SK-BR-3 (both *p* < 0.001) (Figure 3B). BMI influenced activation only in MCF-7 cells, where ATCM from overweight donors enhanced filopodia activation relative to normal weight donors (*p* = 0.030 and *p* = 0.048) (Figure S3B). Thus, naïve adipose tissue in group 1 induced migration of BC cells and this was subtype-dependent. In addition, filopodia activation was seen in all BC cell lines and this can be influenced by BMI.

#### 3.1.2. Group 2—BRCA1/2 Gene Mutation Carrier-Derived Adipose Tissue (BRCA ATCM)

**Figure 4:**
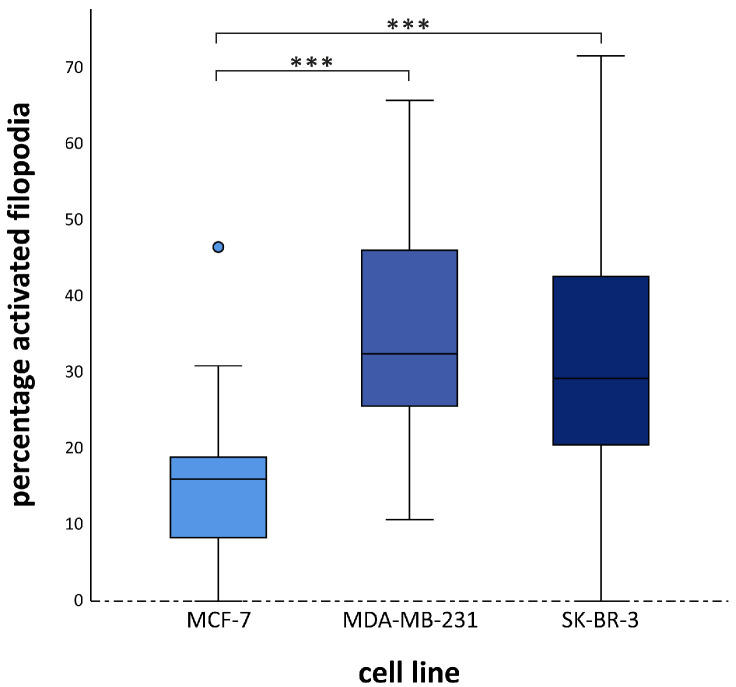
Group 2. Activation of filopodia of MCF-7, MDA-MB-231 and SK-BR-3 cells by BRCA carriers-derived ATCM. BRCA adipose tissue induces filopodia activation. (*** *p* < 0.001) Positive and negative controls are not shown here because these are cell line-dependent and therefore cannot be integrated into this figure.

BRCA1/2 ATCM induced limited migration but significant filopodia activation. Migration was slightly increased in MCF-7 cells, with no effect on MDA-MB-231 or SK-BR-3 cells, and without BMI dependency (Figure S3C). In contrast, filopodia activation increased across all cell lines, with significantly higher activation in MDA-MB-231 and SK-BR-3 compared to MCF-7 (corrected *p* < 0.001 and *p* = 0.001) (Figure 4). BMI effects occurred only in MDA-MB-231 cells, where activation increased progressively with BMI status (NW vs. OB *p* = 0.020; OW vs. OB *p* = 0.004) (Figure S3D). Therefore, while BRCA adipose tissue in group 2 does not generally promote migration, it does enhance filopodia activation in BC cells.

#### 3.1.3. Group 3—Breast Cancer Patient-Derived Adipose Tissue (BC ATCM)

**Figure 5:**
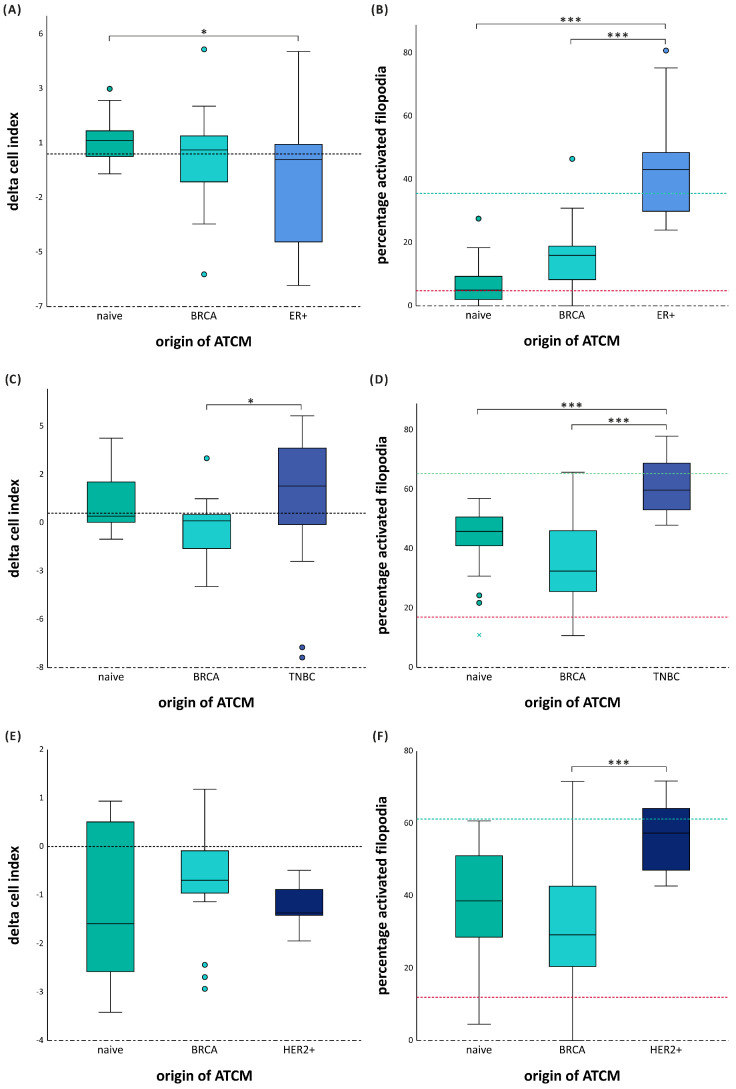
Group 3. Migration and activation induced by BC ATCM. Dotted black line represents zero migration, green line positive control (20% FCS) and red line represents negative control (5% FCS.) for the filopodia activation. ER+ ATCM suppresses migration in MCF-7 cells, it strongly enhances filopodia activation (**A**,**B**). TNBC ATCM both stimulates migration and filopodia activation in MDA-MB-231 cells, producing the strongest pro-invasive response among all groups and subtypes (**C**,**D**). HER2+ ATCM enhances filopodia activation but does not induce migration in SK-BR-3 cells (**E**,**F**). (* *p* < 0.005, *** *p* < 0.001).

ATCM derived from patients with ER+ BC, significantly decreased migration of MCF-7 compared to naïve ATCM (*p* = 0.036), and this effect was independent of BMI (Figure 5A and Figure S3E). In contrast, MCF-7 filopodia activation was strongly increased by ER BC ATCM compared to naïve ATCM and BRCA ATCM (both corrected *p* < 0.001) (Figure 5B). MCF-7 filopodia activation was significantly higher in overweight (*p* = 0.018) and obese (*p* = 0.031) ER+ BC ATCM (Figure S3F). Therefore, although ATCM derived from patients with ER+ BC suppresses migration in MCF-7 cells, it strongly enhances filopodia activation, particularly when BMI increases.

ATCM derived from patients with TNBC, increased both migration and filopodia activation of MDA-MB-231 cells. Migration was significantly increased compared to BRCA ATCM (*p* = 0.026), independent of BMI (Figure 5C and Figure S3G). Similarly, filopodia activation was markedly higher compared to naïve ATCM and BRCA ATCM (corrected *p* = 0.001 and *p* < 0.001), and this effect was independent of BMI (Figure 5D and Figure S3H). Therefore, TNBC BC ATCM simultaneously stimulates migration and filopodia activation in MDA-MB-231 cells, producing the strongest pro-invasive response among all groups and subtypes.

ATCM derived from patients with HER2+ BC, did not increase migration of SK-BR-3 cells, and potential BMI effects could not be assessed due to limited samples. Nevertheless, filopodia activation was significantly increased compared to BRCA ATCM (corrected *p* = 0.007), indicating cytoskeletal activation despite the absence of a migratory response (Figure 5E). Therefore, HER2+ BC ATCM derived from patients with enhances filopodia activation but does not induce migration in SK-BR-3 cells.

In summary, BC derived ATCM in group 3 induced stronger effects on migration and activation than ATCM from healthy subjects or BRCA mutation carriers, in a subtype-specific fashion.

### 3.2. Luminex Assay

ATCM of group 1 and 2 displayed a secretory profile with higher levels of adipokines and metabolic regulators (adiponectin, leptin), as well as Serpin E1 and TNF-α (all corrected *p* < 0.05) compared to group 3. ATCM from group 3 showed significantly elevated levels of cytokines involved in angiogenesis and tissue remodeling (VEGF, PDGF-AA, TGF-α, FGFb), pro-inflammatory signaling (IL-1β, IL-6, IL-8, IL-17, GRO-β, Lymphotoxin-α), immune cell activation and differentiation (CD40, GM-CSF, FLT3L, IFN-α, IL-2, IL-7, IL-12p70, IL-15), and cytotoxic/hematopoietic pathways (Granzyme B, IL-3, IL-5) (all corrected *p* < 0.05) were ATCM of ER+ showed the higher levels. The most pronounced increases were observed for VEGF and PDGF-AA (both corrected *p* < 0.001).

ATCM from ER+ patients showed broad upregulation across angiogenic, pro-inflammatory, and immune-activating cytokines (including IL-6, IL-8, TNF-α, PDGF-AA, VEGF, and PD-L1) compared with ATCM from BRCA 1/2 patients (all corrected *p* < 0.05). In contrast, ATCM from TNBC patients exhibited reduced secretion of inflammatory and angiogenic mediators (G-CSF, IFN-γ, IL-1β, IL-4, IL-6, PDGF-AB/BB, TNF-α) compared with ATCM from ER+ patients, while ATCM from naïve patients displayed higher levels of Serpin E1 and TNF-α relative to ATCM of TNBC patients (all corrected *p* < 0.05). HER2+ ATCM was marked by reduced secretion of the adipokines adiponectin and leptin compared with naïve ATCM (corrected *p* < 0.05) (Table 2).

Overall, obesity was associated with increased secretion of anti-inflammatory mediators. IL-1RA was higher in overweight compared with normal weight samples and further upregulated in obese compared with both normal weight and overweight samples (corrected *p* < 0.05). CRP levels followed a similar pattern, with higher secretion in obese versus normal weight and overweight samples (corrected *p* < 0.05) (Table 3).

## 4. Discussion

In this study we investigated the effect of adipose tissue from different settings on migration and activation of different BC cell subtypes. We showed that even adipose tissue from naïve subjects can induce migration and activation of BC cells in a subtype-dependent matter. In addition, adipose tissue from BRCA 1/2 mutation carriers and BC patients altered or enhanced these results. Cytokine profiling showed elevated levels of angiogenic and inflammatory cytokines in adipose tissue-conditioned media from BC patients. In contrast, non-cancer tissue had higher adipokines like adiponectin and leptin.

In contrast to previous research with co-cultures or adipose tissue stem cells (ASCs) -based conditioned media, in this study the supernatant of patient-derived adipose tissue was used, without extra activation steps [22]. Including patient-derived adipose tissue not only from BC patients but also healthy and BRCA carriers, enabled direct assessment of the effect of adipose tissue from different settings in different BC subtypes. Our findings strengthen the hypothesis that adipose tissue is not an innocent bystander but should be seen as an active player in the BC microenvironment.

The finding that naïve adipose tissue can affect migration of BC cells contributes to the debate on the oncologic safety of lipofilling and autologous fat grafting (AFT). Clinical studies report mixed safety outcomes: Berti et al. show elevated recurrence risk, while Claudio et al. and Navarro et al. found no increased risk when lipofilling occurs >36 months post-surgery or in patients without lymph node involvement [23,24,25]. Reviews generally support this safety but highlight selection bias and methodological limitations [26,27,28]. Due to standard treatments, exclusion of neoadjuvant-treated patients was not feasible. This could indicate treatment-related confounding [29,30,31]. To date, no in vitro studies have examined the effect of adipose tissue from survivors after completion of therapy on BC cells or compared secretion profiles before and after treatment. It is possible that adipose tissue normalizes over time, suggested as the ‘safe interval’ by Silva-Vergara C et al. [25]. However, our pre-clinical findings support continued caution. Identifying markers that distinguish regenerative from pro-tumorigenic signaling will be essential for the safe and personalized use of adipose tissue in breast reconstruction [32].

Filopodia activation was more pronounced in BC ATCM compared to naïve and BRCA ATCM. This observation aligns with prior descriptions that cancer-associated adipocytes and adipocyte-conditioned media promote cytoskeletal remodeling and protrusive activity in BC cells, as shown in several co-culture and ATCM studies [33]. In MCF-7 cells, ER+ ATCM reduced migration but increased filopodia activation, suggesting that activation of migratory machinery does not necessarily translate into movement. This was also previously described by Padilla-Rodriguez et al. [34]. This paradox also reflects the less invasive phenotype of ER+ tumors, consistent with clinical observations and prior studies [33,35,36]. TNBC cells responded strongly to TNBC-matched ATCM, showing increased migration and activation, in line with their aggressive nature and sensitivity to pro-inflammatory adipocyte signals [35,36,37]. BRCA-mutated ATCM did not show these effects, thus genetic background could possibly modulate adipocyte–tumor interactions. In contrast to other subtypes, HER2+ BC cells exhibited little to no migratory response to adipose tissue, and HER2+ ATCM was characterized by reduced adipokine secretion. Together, these findings suggest that HER2+ BC cells are less sensitive to adipose tissue-derived signals, consistent with HER2 signaling being the dominant oncogenic driver in this subtype.

Most of the previous work about interaction of adipose tissue and BC cells are BMI orientated. In this study we did not find strong BMI dependency in migration behavior. We did see that higher BMI was associated with increased filopodia activation in ER+ cells and increased secretion of cytokines (IL-1RA, CRP) which are markers of inflammation, in line with literature that show a chronic pro-tumorigenic microenvironment in obesity [33,37,38,39,40]. CRP has also shown to be associated with bad prognosis in BC [41]. These findings align with previous studies showing that adipocytes can promote BC progression via cytokines such as IL-6, leptin, and VEGF, with obesity amplifying these effects [33,40,42,43]. Although this points to BMI as the culprit, this could be more dependent on the metabolic state of the adipose tissue. Growing literature shows that adipose tissue can be ‘unhealthy’ in normal weight patients and ‘healthy’ in obese patients, suggesting that BMI might not always directly align with the metabolic activity of adipose tissue [44,45]. In the context of our results, this illustrates the need to identify better markers of metabolic activity of BC patients’ adipose tissue in the future.

This study has limitations. Patient samples of adipose tissue contain more than only adipocytes. There could be other cells or aliquots within the adipose tissue that could have influenced the results. Another limitation includes the small sample sizes, particularly in the HER2+ subgroup, reducing statistical power. Additionally, we could not fully exclude patients receiving neoadjuvant therapy, which may alter adipose characteristics [30,31]. On the other hand, neoadjuvant treatment is the mainstay treatment for HER2 positive BC and TNBC, and therefore these samples do reflect current clinical practice. Future studies should refine this model by culturing adipose-derived stem cells directly from adipose tissue and analyzing their secretion pattern, or by employing co-culture systems to better mimic the cellular interactions within the tissue environment [33,36,46].

## 5. Conclusions

This study shows that breast adipose tissue from healthy women, BRCA 1/2 mutation carriers and BC patients, can stimulate BC cell line migration and activation. This effect is related to BC subtype and BMI. These data improve insight in adipose tissue as factor in BC development.

## Figures and Tables

**Table 1:** Patient characteristics.

	Naïve	BRCA Mutation	ER+ BC	TNBC BC	HER2+ BC
BMI * mean ± SD (*n*)	26.7 ± 4.3 (*n* = 20)	27.0 ± 3.8 (*n* = 22)	32.4 ± 3.3 (*n* = 19)	27.7 ± 6.3 (*n* = 12)	26.8 ± 5.1 (*n* = 7)
Normal weight ^1^	22.9 ± 2.0 (*n* = 9)	22.6 ± 1.8 (*n* = 7)	23.4 ± 1.2 (*n* = 6)	21.8 ± 1.8 (*n* = 4)	21.5 ± 1.6 (*n* = 3)
Overweight ^2^	27.2 ± 1.1 (*n* = 5)	27.5 ± 1.5 (*n* = 8)	27.3 ± 1.6 (*n* = 9)	28.0 ± 0.8 (*n* = 5)	29.7 (*n* = 1)
Obese ^3^	31.9 ± 2.3 (*n* = 6)	31.0 ± 1.9 (*n* = 7)	37.0 ± 1.1 (*n* = 4)	35.0 ± 7.7 (*n* = 3)	31.1 ± 1.0 (*n* = 3)
age	34 ± 14	33 ± 9	57.3 ± 14	49 ± 15	58 ± 15
Neo-adjuvant treatment (%)	0%	0%	0%	30.7%	14.3%

* Body mass index, ^1^. BMI ≤ 25 normal weight, ^2^. 25 < BMI > 30 overweight and ^3^. BMI ≥ 30 obese.

**Table 2:** Luminex assay naïve versus BC adipose tissue.

Marker/Cytokine	Highest Sample	Test Statistics	SE	SD	*p* (Corrected)
CD40	BC	1051.5	103.8	2.4	0.015
FGF b	BC	1019.0	103.8	2.1	0.033
FIT3_L	BC	1053.0	103.8	2.5	0.014
GM_CSF	BC	1111.5	103.8	3.0	0.003
GranymeB	BC	1010.5	103.8	2.0	0.041
GRO_B	BC	1028.0	103.8	2.2	0.027
IFN_alfa	BC	1012.5	103.8	2.1	0.039
IL12p70	BC	1031.5	103.5	2.3	0.024
IL15	BC	1019.5	103.7	2.1	0.033
IL17	BC	1070.0	102.9	2.6	0.008
IL1B	BC	1068.0	103.3	2.6	0.009
IL1RA	BC	1022.0	103.8	2.2	0.031
IL2	BC	1129.5	103.8	3.2	0.001
IL3	BC	1093.5	103.7	2.9	0.004
IL5	BC	1012.0	99.4	2.2	0.031
IL6	BC	1052.5	103.8	2.5	0.014
IL7	BC	1031.5	103.7	2.3	0.024
IL8	BC	1122.0	103.8	3.1	0.002
Lymfotoxin_a	BC	1079.0	103.6	2.7	0.007
PDGF_AA	BC	1161.0	103.8	3.5	<0.001
TGF a	BC	1069.5	103.6	2.6	0.009
VEGF	BC	1143.5	103.8	3.3	<0.001
adiponectin	Non BC	400.0	101.3	−3.9	<0.001
Leptin	Non BC	200.0	103.8	−5.8	<0.001
Serpin E1	Non BC	584.0	584.0	−2.1	0.037
TNF-Alfa	Non BC	1331.0	590.0	−2.0	0.045

**Table 3:** Luminex assay shows differences in cytokine presence in adipose tissue between different BMI groups.

Marker/Cytokine	Samples *	Test Statistics	SE	SD	*p* (Corrected)
IL1RA	NW-**OW**	−16.8	6.1	−2.8	0.018
	NW-**OB**	−20.4	6.5	−3.1	0.005
CRP	NW-**OB**	−18.0	6.5	−2.8	0.017
	OW-**OB**	−17.2	6.6	−2.6	0.029

* Bold sample contained the highest cytokine presence.

## Data Availability

The datasets generated and/or analyzed during the current study are available from the corresponding author on reasonable request.

## Supplementary Materials

The following supporting information can be downloaded at: https://www.mdpi.com/article/10.3390/cancers18050868/s1, Method S1: ATCM Preparation Protocol; Method S2: Detailed Migration Assay Protocol and CI Correction; Method S3: Detailed Scratch Assay and Filopodia Quantification; Figure S1: An overview of the preparation of the ATCM (adipose tissue controlled medium).; Figure S2: Migration data for timepoints 24 hours in the top row and 48 hours in the bottom row. A; for naïve ATCM (group1), B; BRCA ACTM (group2) and C–E (group 3); C; MCF-7, D; MDA-MB-231 and E; SK-BR-3.; Figure S3: Migration and filopodia activation subdivided by BMI for group 1(A & B), group 2 (C & D) and group 3 (E–H). BMI groups are NW = normal weight (BMI < 25), OW = overweight (25 ≤ BMI < 30). OB=obese (BMI ≥ 30) which are shown from light to darker pink. Dotted black line represents zero migration, green line positive control (20% FCS) and red line represents negative control (5% FCS.) for the filopodia activation. (* *p* < 0.005, ** *p* = 0.001, *** *p* < 0.001) [21,47,48].

## Abbreviations

The following abbreviations are used in this manuscript:

BC	Breast cancer
ATCM	adipose tissue conditioned medium
BMI	Body Mass Index
PBS	phosphate-buffered saline
DMEM	Dulbecco’s Modified Eagle Medium
RTCA	Real-Time Cell Analysis
NW	Normal weight (BMI < 25)
OW	Overweight (25 ≤ BMI < 30)
OB	Obese (BMI ≥ 30)
