# Supplementary material for: Effect of Healthy and Tumor-Associated Breast Adipose Tissue on Breast Cancer Cell Migration and Activation
Source: Cancers (Basel). 2026 Mar 8;18(5):868. doi: 10.3390/cancers18050868 (PMC12985100; doi:10.3390/cancers18050868)
Supplement: Supplementary file 1 [file cancers-18-00868-s001.zip › cancers-4175122-supplementary.pdf]

Supplementary materials

Effect of Healthy and Tumor-Associated Breast Adipose Tissue on Breast Cancer Cell Migration and Activation

Iris L. Holt-Kedde, Hetty Timmer-Bosscha, Frank A. E. Kruyt, Wendy Kelder, Bert van der Vegt, Mieke C. Zwager , Carolien P. Schröder and Marlous Arjaans

Method S1 – Detailed ATCM Preparation Protocol

Preparation of human ATCM was based on the previous description by Wan et al. [1], with some modifications. Up to four fresh adipose tissue samples of approximately 1 cm^3 were selected per patient by the Department of Pathology. Samples were transported from the Department of Pathology in sterile PBS on ice and processed within 1–2 hours.

Tissues were washed three times in PBS to remove blood and debris. Macroscopically visible blood vessels and glandular components were carefully dissected and discarded. The remaining adipose tissue was minced into fragments of approximately 1–2 mm in diameter.

Ten tissue fragments were transferred to each T75 culture flask containing 10 mL Dulbecco's Modified Eagle Medium low glucose (DMEM-Low, Invitrogen) supplemented with 1% fetal calf serum (FCS) and 1% penicillin-streptomycin. This ratio was determined in pilot studies to balance tissue viability with sufficient release of soluble factors. Flasks were incubated at 37°C and 5% CO_2 for 48 hours under sterile conditions.

After incubation, ATCM was collected, centrifuged to remove residual debris, and aliquoted into sterile 1 mL cryogenic vials. All aliquots were stored at –80°C until further use in functional assays.

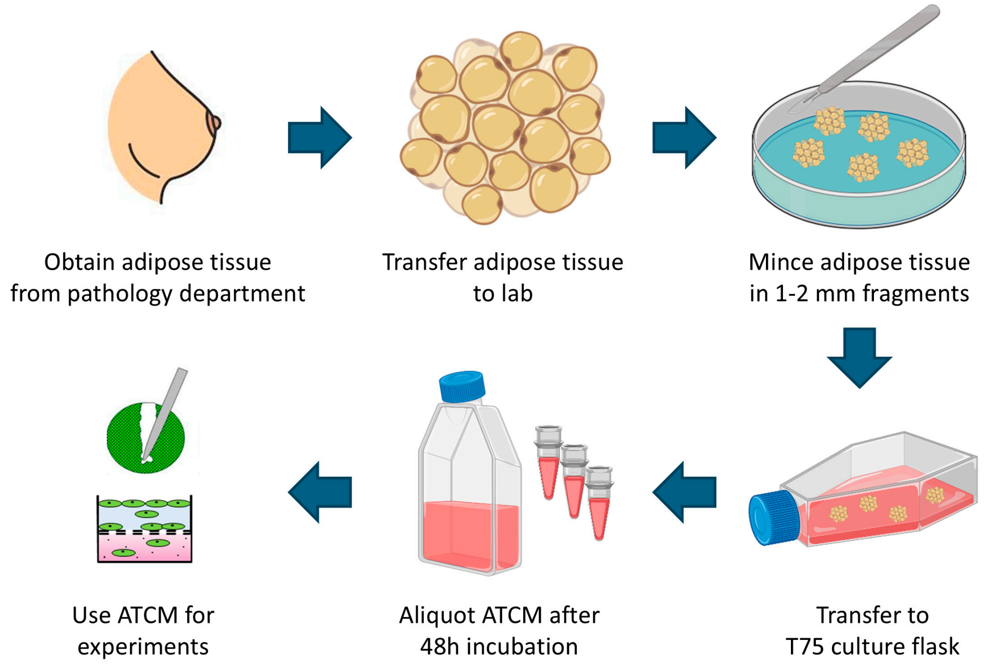

Figure S1. An overview of the preparation of the ATCM (adipose tissue controlled medium).

Method S2 – Detailed Migration Assay Protocol and CI Correction

Real-time monitoring of BC cell migration was performed using the xCELLigence Real-Time Cell Analysis Dual Plate (RTCA DP) system (Agilent Technologies) with a

CIM-Plate 16 containing upper and lower chambers separated by an 8 μm microporous polyethylene terephthalate membrane. Migration was measured as cell index (CI), derived from impedance changes detected by microelectronic sensors integrated into the underside of the membrane.

Cells were seeded at 5×10^4 cells/well in DMEM-Low containing 1% FCS in the upper chamber. The lower chamber was filled with ATCM from the three study groups. For groups 1 and 2, all three cell lines were tested; group 3 was only tested with the subtype-matched cell line.

Negative control consisted of DMEM-Low with 1% FCS and positive control consisted of DMEM with 20% FCS. Migration was recorded every 10 minutes for up to 60 hours.

To ensure comparability between assays, CI values were corrected for positive and negative controls using the following formula:

$$\frac{(\text{CI sample} - \text{CI negative control})}{(\text{CI positive control} - \text{CI negative control})} = \text{delta cell index}$$

A delta cell index near 0 corresponds to the negative control and near 1 to the positive control; values below 0 or above 1 indicate reduced or enhanced migration, respectively.

Migration was evaluated at 24, 36, and 48 hours. The 36-hour time point was selected as optimal, as migration was still increasing at 24 hours and measurements at 48 hours could be influenced by proliferation-related effects.

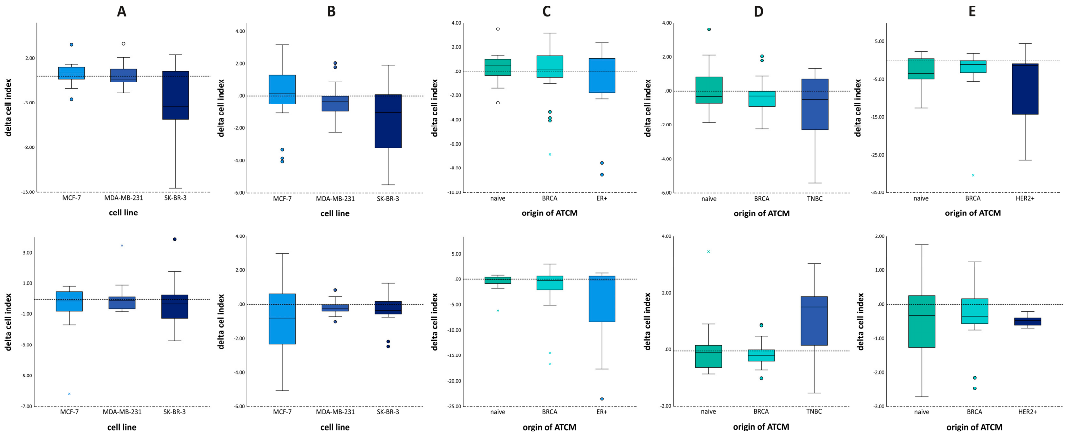

Figure S2. Migration data for timepoints 24 hours in the top row and 48 hours in the bottom row. A; for naïve ATCM (group1), B; BRCA ACTM (group2) and C-E (group 3); C; MCF-7, D; MDA-MB-231 and E; SK-BR-3.

Method S3 – Detailed Scratch Assay and Filopodia Quantification

Cells were seeded on coverslips (ø13 mm, thickness 1, VWR) inserted in 24-well plates (Costar® 3524). For MCF-7 cells, coverslips were coated with poly-L-lysine to maintain a confluent layer. Seeding densities were as follows: 1×10^5 cells for MCF-7, 7.4×10^5 for MDA-MB-231, and 1.5×10^5 for SK-BR-3 in 1 mL medium to achieve ~70% confluence after 24 hours.

Following 24-hour starvation, a scratch was made using a 1 mL pipette tip. Cells were treated with 400 μL ATCM from groups 1–3 (group 3 matched to BC subtype). Controls were included as described above.

After 24 hours, cells were fixed with 4% methanol-free formaldehyde and permeabilized using 0.1% Triton. F-actin was stained with phalloidin (1:40 from 40X methanolic stock solution, Thermo Fisher Scientific #A22283), and nuclei were stained with DAPI (1:200, Sigma D-9542).

Slides were analyzed using an Axio Imager 2 microscope with Zen 3.0 software (Zeiss). For each slide, three overview images (100× magnification) were acquired. From these, six high-power fields (HPFs) along the scratch were captured at 400× magnification. Where possible, two HPFs per overview image were taken from opposite sides of the scratch.

Images were analyzed using Fiji ImageJ version 2.9.0. Filopodia were identified according to previously described methods [2,3], with modifications. Quantification was performed in greyscale, and activated filopodia were identified based on phalloidin staining intensity using the Fire lookup table (Figure 2). Activation was reported as the percentage of positive filopodia relative to total filopodia.

Filopodia were manually identified according to the following criteria:

- Structures cut off at the HPF edge were excluded
- Must extend ≥ 4 μm from the cell edge
- Must be ≥ 3 μm wide at the base
- Structures ≥ 10 μm apart were counted as separate filopodia
- Filopodia did not exceed the length of one cell
- All images were acquired using identical microscope settings.

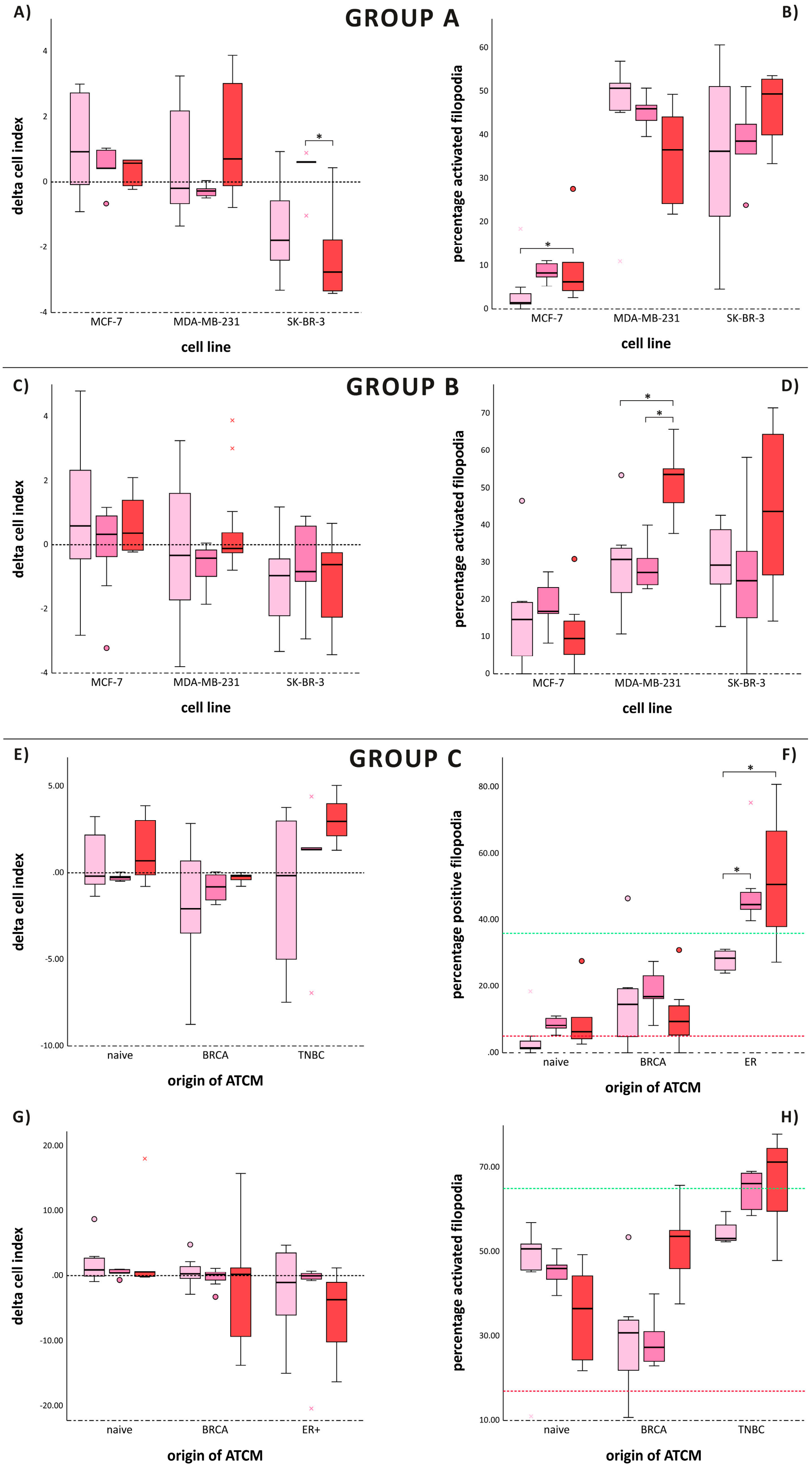

Figure S3. Migration and filopodia activation subdivided by BMI for group 1(A & B), group 2 (C & D) and group 3 (E-H). BMI groups are NW=normal weight (BMI<25), OW=overweight (25≤BMI<30). OB=obese (BMI≥30) which are shown from light to darker pink. Dotted black line represents zero migration, green line positive control (20% FCS) and red line represents negative control (5% FCS.) for the filopodia activation. (*p<0.005, **p=0.001, ***p<0.001).

Reference

1 Cells, S.N.; Wan, Z.; Mah, D.; Simtchouk, S.; Kluftinger, A. Human Adipose Tissue Conditioned Media from Lean Subjects Is Protective against H_2O_2 Induced Neurotoxicity in Human SH-SY5Y Neuronal Cells. *Int. J. Mol. Sci.* **2015**, *16*, 1221–1231. https://doi.org/10.3390/ijms16011221.

2 Bischoff, M.C.; Lieb, S.; Renkawitz-Pohl, R.; Bogdan, S. Filopodia-based contact stimulation of cell migration drives tissue morphogenesis. *Nat. Commun.* **2021**, *12*, 791. https://doi.org/10.1038/s41467-020-20362-2.

3 Nilufar, S.; Morrow, A.A.; Lee, J.M.; Perkins, T.J. FiloDetect: Automatic detection of filopodia from fluorescence microscopy images. *BMC Syst. Biol.* **2013**, *7*, 66. https://doi.org/10.1186/1752-0509-7-66.
